# Charge compensation for NADPH oxidase activity in microglia in rat brain slices does not involve a proton current

**DOI:** 10.1111/j.1460-9568.2008.06417.x

**Published:** 2008-09

**Authors:** Anna De Simoni, Nicola J Allen, David Attwell

**Affiliations:** Department of Physiology, University College LondonGower Street, London WC1E 6BT, UK

**Keywords:** membrane current, microglia, NADPH oxidase

## Abstract

The membrane properties of isolated cultured microglia have been extensively studied but it is important to understand their properties *in situ*, where they protect the brain against infection, but also contribute to neurodegenerative diseases. Microglia and macrophages attack bacteria by generating reactive oxygen species, a process which involves NADPH oxidase pumping electrons out across the cell membrane. The resulting inward current evokes a depolarization, which would inhibit the activity of the NADPH oxidase if there were no charge-compensating current which moves positive charge out across the membrane. The mechanism of this charge compensation is controversial. In neutrophils and in cultured microglia a depolarization-activated H^+^ conductance has been proposed to provide charge compensation, and also to remove protons generated intracellularly by the NADPH oxidase. Alternatively, a depolarization-activated K^+^ conductance has been proposed to mediate charge compensation. Here we show that in microglia, either in the resting state or when activated by the bacterial coat component lipopolysaccharide, both in acute and in cultured hippocampal slices, no significant H^+^ current is detectable. This implies that the membrane properties of microglia in their normal cellular environment differ from those of cultured microglia (similarly, microglia generated a current in response to ATP but, unlike in culture, not to glutamate or GABA). Furthermore, the K^+^ current (Kv1.3) that is activated by lipopolysaccharide is inactivated by depolarization, making it unsuitable for mediating charge compensation on a long time scale at positive voltages. Instead, charge compensation may be mediated by a previously undescribed non-selective cation current.

## Introduction

Microglia, the immune cells of the brain, constantly survey the brain microenvironment and respond to infection or injury. The membrane properties of microglia are fundamental to their surveillance of the brain parenchyma. In pathological states, brain microglia are activated to produce a ‘respiratory burst’, which generates reactive oxygen species (ROS), in particular superoxide and peroxynitrite, that cause the death of target cells (Demaurex & Petheo, 2005). However, in neurodegenerative conditions such as Alzheimer’s and Parkinson’s diseases, HIV and prion infection, and multiple sclerosis, ROS generated by microglia contribute to the death of neurons (reviewed by Block *et al.*, 2007). ROS are produced as a result of the microglial enzyme NADPH oxidase expelling electrons across the cell membrane. In this process, H^+^ accumulates inside microglia and the cells are depolarized by the electron current. As excessive depolarization inhibits further expulsion of electrons (DeCoursey *et al.*, 2003), a charge-compensating mechanism is required to limit the depolarization of the microglial cells and thus to maintain the activity of the NADPH oxidase.

Controversy exists about the conductance responsible for charge compensation (DeCoursey, 2004; Segal, 2005; Femling *et al.*, 2006). A depolarization-activated proton current suitable for this role has been described in eosinophils, neutrophils and cultured microglia (DeCoursey & Cherny, 1993; Eder *et al.*, 1995; Visentin *et al.*, 1995; McLarnon *et al.*, 1997; Cherny *et al.*, 2001; Eder & DeCoursey, 2001). This would also prevent cytoplasmic acidification by removing the H^+^ generated by the NADPH oxidase. Alternatively, a K^+^ efflux through Ca^2+^-activated K^+^ channels has been advocated to provide a current to balance the charge movement generated by the NADPH oxidase activity, and in addition to activate the release of bacteriocidal proteases (Reeves *et al.*, 2002; Ahluwalia *et al.*, 2004; Segal, 2005).

A detailed study has demonstrated that proton channels, but not K^+^ channels, are required for the antibacterial activity of neutrophils and eosinophils (Femling *et al.*, 2006). In cultured microglia, however, the voltage-gated K^+^ conductance Kv1.3 and the Ca^2+^-activated K^+^ conductances SK2 and SK4 are necessary for the respiratory burst and peroxynitrite formation, and inhibiting Kv1.3 prevented neuronal killing by the microglia (Khanna *et al.*, 2001; Fordyce *et al.*, 2005), suggesting that voltage-gated K^+^ channels may provide charge compensation for the NADPH oxidase current in cultured microglia. Finally, Thomas *et al.* (2007) have suggested that Cl^−^ channels (possibly activated by swelling: Schlichter *et al.*, 1996; Eder *et al.*, 1998) may also contribute to charge compensation in cultured microglia.

There have been only a few studies of the electrophysiological properties of microglia *in situ* in brain slices where, unlike cultured microglia, they are surrounded by neurons and astrocytes (Brockhaus *et al.*, 1993; Boucsein *et al.*, 2000, 2003; Bordey & Spencer, 2003). These earlier studies did not establish the membrane currents responsible for charge compensation of the NADPH oxidase electron flux. As isolated cultured cells often differ in their properties from cells *in situ*, we have examined the membrane currents of microglia in hippocampal slices, focusing on those which might mediate charge compensation.

## Materials and methods

### Acute slice preparation

Sprague Dawley rats at postnatal day 21 were killed by cervical dislocation in accordance with UK animal experimentation regulations. Animals were then decapitated and 250-μm hippocampal slices were cut using a vibrating slicer. Slice preparation was carried out in the bicarbonate buffered solution described below, with kynurenic acid (1 mm, Sigma, UK) added (to block glutamate receptors to reduce potential excitotoxic damage during slicing).

### Organotypic slice preparation

Organotypic slices were prepared using the method of Stoppini *et al.* (1991) as described previously (De Simoni & Yu, 2006). Animals were killed by cervical dislocation in accordance with UK animal experimentation regulations. In brief, 300-μm thick parasagittal hippocampal slices were prepared from 7-day-old male rat pups under sterile conditions in a laminar flow cabinet using a vibrating slicer. Each slice was placed on a filter in the bottom of a Millicell culture plate (Millipore U.K. Ltd, UK). The slices were at an interface with serum culture medium comprising: 25% horse serum, 50% minimal essential medium (MEM), 23% Earle’s balanced salts solution (EBSS) (all from GIBCO BRL, UK), 5000 U/100 mL penicillin (0.08 mm) and 1200 U/100 mL nystatin (both from Sigma). The medium was changed three times per week. Individual slices were removed from the incubator for experiments on the appropriate day *in vitro* (DIV).

### Microglial cell labelling

To label microglia, slices were incubated for 20 min in 1 mL of external solution containing isolectin-B4 conjugated to Alexa 488 (Molecular Probes, UK: excited at 475 nm and emission collected at 535 nm) at a concentration of 50 μg/mL (Streit & Kreutzberg, 1987).

### Extracellular solutions

Patch-clamp recordings were obtained from fluorescently visualized cells at 33°C, in bicarbonate- or HEPES-buffered solutions. Bicarbonate-buffered solution contained (mm): 126 NaCl, 24 NaHCO_3_, 1 NaH_2_PO_4_, 2.5 KCl, 2.5 CaCl_2_, 2 MgCl_2_, 10 glucose (gassed with 95%O_2_/5% CO_2_), pH 7.4. HEPES-buffered solution was used when a change in the external pH was required, and contained (mm): 144 NaCl, 10 HEPES, 1 NaH_2_PO_4_, 2.5 KCl, 2.5 CaCl_2_, 2 MgCl_2_, 10 glucose (gassed with 100% O_2_). When the pH was increased to 8.5, TAPS buffer was sometimes used instead of HEPES (the results were indistinguishable from when HEPES was used as the buffer). When clotrimazole (Sigma) was added to the external solution from a stock solution in dimethyl sulfoxide (DMSO), a corresponding amount of DMSO was added to the control solution. Flufenamic acid (Sigma, UK) was made up as a stock in ethanol. LaCl_3_ (BDH Chemicals Ltd, UK) was prepared as a stock solution in water. To activate microglia, bacterial lipopolysaccharide (LPS, 1 μg/mL; Sigma), which may mimic infections contributing to the causes of cerebral palsy, was added to the external solution of acute slices for up to 8 h before recording, and to the culture medium of organotypic slices 24 h before recording.

### Electrophysiology

Recordings were performed by whole-cell patch-clamping, using patch pipettes pulled from thick-walled borosilicate glass capillaries and filled with an internal solution containing (mm): 130 KCl, 4 NaCl, 10 HEPES, 10 EGTA, 2 MgATP, 0.5 NaGTP, 6 NADPH, pH adjusted to 7.2 with KOH. Alexa 568 (0.2 mg/mL; Molecular Probes) was also included to verify the targeted cell had been successfully recorded from, and to identify cell morphology. For experiments using low Cl^−^ internal solution, the solution contained (mm): 130 Cs-gluconate, 4 NaCl, 10 HEPES, 10 BAPTA, 4 MgATP, 0.5 NaGTP, 0.5 CaCl_2_, pH adjusted to 7.2 with CsOH. The whole-cell series resistance was ∼20 MΩ before compensation by 50% to reduce it to ∼10 MΩ. Some experiments employed ADP-ribose (Sigma) in the internal solution to activate TRPM2 (transient receptor potential melastatin2) channels. Data were sampled at 10 kHz and filtered at 2 kHz. Electrode junction potentials were compensated for.

### Confocal microscopy and ROS measurement

Isolectin-B4-labelled microglia were counted after imaging by a Pascal confocal scanhead (Zeiss) based on an upright Axioskop 2 microscope (Zeiss), using a 63× water-immersion objective with numerical aperture of 0.95. A stack of image planes separated by 0.5 μm was acquired, composed of 40 optical sections (512 × 512 pixel arrays, two scans averaged per optical section) spanning 20 μm in the vertical (*z*) dimension. The imaged area (146 × 146 μm) was within the stratum radiatum of hippocampal area CA1. The imaging chamber was continuously perfused with bicarbonate-buffered solution at 33°C (gassed with 95% O_2_–5% CO_2_).

Cellular production of ROS was visualized through the O_2_-specific oxidation of dihydroethidium to ethidium, which binds to the DNA of O_2_-producing cells. Dihydroethidium (5 μm; Sigma) was added to all solutions. No preincubation was used, to limit the intracellular accumulation of oxidized product. Changes in the rate of rise of the signal (averaged over 200 s) were measured to obtain changes in rates of ROS generation. Ethidium fluorescence was excited at 543 nm, and measured at 560 nm, while isolectin-B4 was imaged using excitation at 488 nm and emitted fluorescence was collected at 505–600 nm, using a Pascal confocal scanhead (Zeiss) based on an upright Axioskop 2 microscope (Zeiss), using a 63× water-immersion objective with numerical aperture of 0.95 (theoretical spatial resolution ∼0.3 μm). Images were taken every 5 s.

### Statistics

Data are presented as mean ± SEM. The significance of changes was assessed with Student’s two-tailed *t*-test or one-way anova, at the 95% confidence level.

## Results

### Choice of preparation

To study microglia in their native environment, surrounded by neurons and astrocytes, we used hippocampal slices. Microglia in freshly made (‘acute’) slices are less likely than isolated cultured microglia to have dramatically changed their properties. We used slices from postnatal day (P)21 rats, so that microglia will have returned to a resting state after the period of neuronal death early in development. In the hours after slicing, some microglia become activated by the slicing process [Stence *et al.* (2001), although cells can stay ramified for hours: see below and Boucsein *et al.* (2000)], so we also compared the properties of microglia in acute slices with those of microglia in organotypic cultured slices. In organotypic slices, after 1–2 weeks in culture the microglia activated by the slicing revert to a resting state (Hailer *et al.*, 1996). The use of organotypic slices also allowed us to activate the cells in a controlled way by applying LPS for 24 h (which was not possible in acute slices as they do not survive for 24 h). As detailed below, we observed no difference in the electrophysiological properties of the microglia in the two preparations (apart from an upregulation of K^+^ current induced by LPS).

### Morphology of resting and activated microglial cells in acute and organotypic slices

**Fig. 1:**
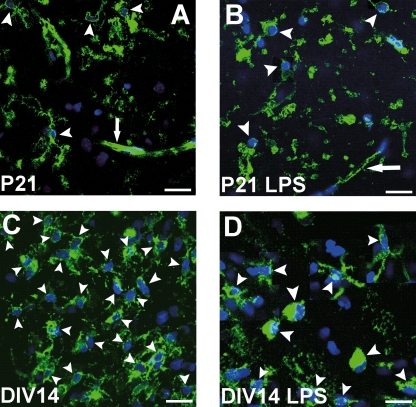
Morphology of microglia in acute and organotypic hippocampal slices. (A) Isolectin-B4-labelled microglial cells (arrowheads) in P21 acute slices. (B) After 8 h incubation with LPS (1 μg/mL), cells in P21 acute slices displayed a similar morphology. The arrows indicate labelled blood vessels. (C) Microglia in DIV 14 organotypic slice. Cells assumed a resting state, with long and thin processes and a small soma. (D) After 24 h incubation with LPS, macrophage-like microglial cells appeared in organotypic slices. Scale bar = 20 μm. All images are from the CA1 stratum radiatum, each projected stack being composed of 40 scans of 0.5 μm, giving a total depth of 20 μm.

Microglial cells in P21 acute slices were labelled with isolectin-B4 and imaged live with a confocal microscope, 3–8 h after slicing. The different areas of the hippocampus (CA1, CA3 and dentate gyrus) showed a similar density and morphology of microglia. Some cells appeared ramified, while others had a small number of retracted, coarse branches and an enlarged soma (Fig. 1A, from stratum radiatum), in agreement with previous reports from acute slices (Dalmau *et al.*, 1998; Boucsein *et al.*, 2000). Incubation for up to 8 h with LPS (1 μg/mL) did not affect the mean soma diameter of microglia, which was 6.74 ± 0.45 μm (*n* = 11) in control conditions and 6.59 ± 0.26 μm (*n* = 27) after LPS (not significantly different, p = 0.78), but slightly reduced the number of processes per cell, which was 3.64 ± 0.39 (*n* = 11, defined as the number of processes leaving the soma) in control conditions and 2.78 ± 0.20 (*n* = 27) after LPS (significantly reduced, *P* = 0.038). Their density (per 146 × 146-μm area, including cells at the surface of the slice) was not significantly affected, being 3.7 ± 0.3 in control conditions (eight areas from four slices) and 4.5 ± 0.4 after 6 h of LPS (six areas from three slices; not significantly different, *P* = 0.16). Isolectin-B4 also labels blood vessels, as seen in Fig. 1A and B.

When imaged in organotypic slices after 14–15 DIV, many microglial cells assumed a typical resting morphology with a round soma and ramified processes (Fig. 1C), similar to that observed in cultured slices by Duport & Garthwaite (2005). The morphology of cells was homogeneous within the different regions (CA1, CA3, DG), except at the edges of the slice, where microglia assumed an amoeboid shape as described by Duport & Garthwaite (2005). Moreover, in agreement with Duport & Garthwaite (2005), the cell density was higher than in acute preparations: in the absence of LPS the cell density (per 146 × 146-μm area, including cells at the surface of the slice) was 26.8 ± 3.9 (five areas from four slices; *P* = 0.0001 compared with acute slices). After 24 h incubation with LPS (1 μg/mL) some cells became more rounded and retracted their processes (Fig. 1D), and they migrated onto the top of the slice. The cell density was reduced by LPS to 13.6 ± 1.1 cells per 146 × 146-μm area (six areas in three slices; *P* = 0.007 compared with no LPS), consistent with LPS inhibiting microglial cell division (Ganter *et al.*, 1992). The mean soma diameter was 9.07 ± 0.24 μm (*n* = 134) in control conditions (significantly larger than in acute slices, *P* = 0.007) and 9.96 ± 0.29 μm (*n* = 82) after LPS (significantly increased, *P* = 0.021). The number of processes per cell was 2.36 ± 0.12 (*n* = 134) in control conditions (significantly lower than in acute slices, *P* = 0.004), and was not affected significantly by LPS (2.10 ± 0.15, *n* = 82, *P* = 0.17).

### Membrane properties of microglia

**Fig. 2:**
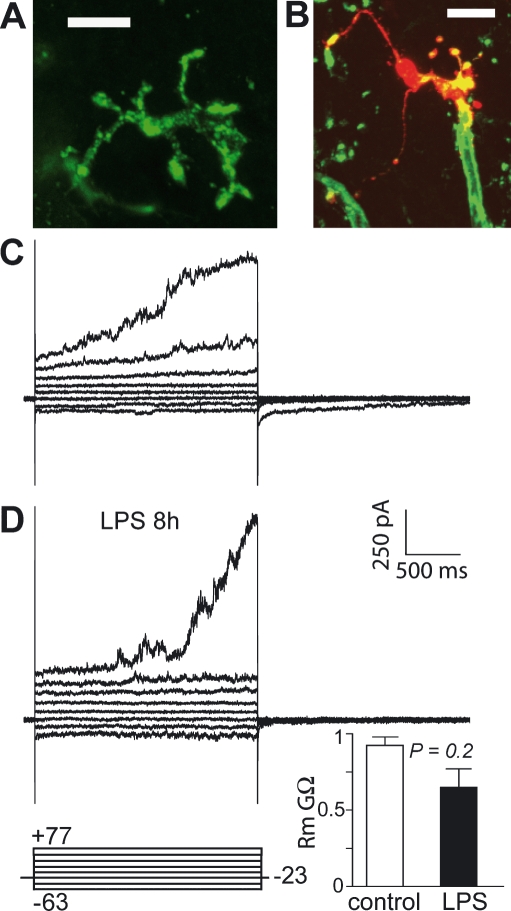
Membrane currents in microglia in acute slices. (A) High-power view of a hippocampal microglial cell labelled with isolectin-B4 in an acute slice; (B) similar cell after whole-cell clamping with Alexa 568 introduced from the recording pipette. Scale bars = 10 μm. (C) Current evoked by 20 mV steps (inset at bottom) from a holding potential of −23 mV, in a P21 acute slice. Note the outward current developing at potentials positive to +40 mV. (D) After incubation with LPS (1 μg/mL), the membrane current in microglia from P21 acute slices did not differ from control. The inset shows no significant difference in the membrane resistance (at −23 to −43 mV) in microglia from control (119 cells) and LPS treated slices (nine cells).

Figure 2A shows a higher power view of a hippocampal microglial cell labelled with isolectin-B4 in an acute slice, and Fig. 2B shows a similar cell after whole-cell clamping with Alexa 568 introduced from the recording pipette.

**Fig. 3:**
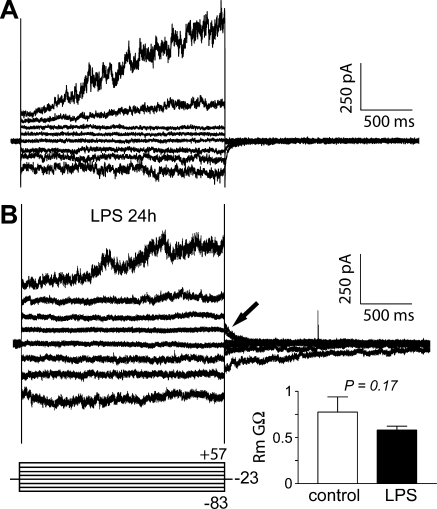
Membrane currents in microglia in organotypic slices. (A) Current evoked by 20 mV steps (inset at bottom) from a holding potential of −23 mV, in a microglial cell in an organotypic slice. (B) After 24 h incubation with LPS (1 μg/mL), microglia in organotypic slices showed a current that recovered from inactivation during negative voltage steps and then produced an outward current tail on repolarization to −23 mV (arrow). No difference was found in the current activated at potentials positive to +40 mV compared with control slices not exposed to LPS. The inset shows the membrane resistance (at −23 to −43 mV) in control (11 cells) compared with LPS-treated slices (18 cells), with no significant difference.

Cells were voltage-clamped at a holding potential of −23 mV, as membrane currents became unstable and the seal was lost within a few minutes if a more negative holding potential near the K^+^ equilibrium potential was used, as found previously by Boucsein *et al.* (2000). Membrane currents were recorded in response to voltage steps of 20 mV lasting 2 s, from −83 to +77 mV (acute slice, Fig. 2C; organotypic slice, Fig. 3A). Membrane resistance was measured from the last 100 ms of the response to a step from −43 to −23 mV, and was not significantly different (*P* = 0.44) in acute (Fig. 2 inset, 0.92 ± 0.06 GΩ, *n* = 119) and organotypic slices (Fig. 3 inset, 0.78 ± 0.16 GΩ, *n* = 11), despite the fact that the microglia in organotypic slices should be more in a resting state than in the acute slices. In 145 cells in acute slices cell capacitance was 15.0 ± 0.4 pF, while in 22 cells in organotypic slices it was 15.6 ± 1.8 pF (not significantly different, *P* = 0.59).

The resting membrane potential of microglia, as calculated from the zero current potential of the *I–V* relationship (which includes shunting by the seal resistance), was −18 ± 1 mV measured in 106 cells in acute slices. In a subset of 26 cells, for which the seal resistance was measured before going to whole-cell mode, correcting for the seal conductance (assumed to have a reversal potential of 0 mV) as in Tessier-Lavigne *et al.* (1988) suggested that the true resting potential is roughly 2.3 times these values, around −41 mV, and the true membrane resistance is also 2.3-fold larger than the measured value, i.e. around 2.1 GΩ in acute slices.

Although cells showed an ohmic *I–V* relationship over the voltage range from −80 to 0 mV, at potentials positive to about +20 mV an outward current started to develop, with an amplitude that increased with depolarization and with time, which is discussed in detail below. This current frequently did not increase smoothly with time, showing periods of accelerated activation after a delay (e.g. Fig. 2C and D) and on repeated depolarization to the same potential the current activated with a somewhat different time course each time (see Fig. 7A). Upon returning to −23 mV after a depolarizing step, inward current tails appeared in control conditions (Figs 2C and 3A), showing that the reversal potential of the current activated by depolarization was more positive than −23 mV (the decaying inward current tails reflect deactivation of the current that was activated by the preceding depolarizing pulse). The amplitude of the increase in outward current at positive potentials and the time course of the subsequent inward tail at −23 mV (which are quantified below) showed significant variability both between cells and on repeated depolarization of the same cell, but on average were not significantly affected by LPS (see below).

### The response of microglia to neurotransmitters

To investigate the possibility that transmitter release, for example in ischaemia, might be a trigger for the activation of resting microglia, a variety of agents activating neurotransmitter receptors were applied to ramified microglia in acute slices while applying voltage steps to various potentials from a holding potential of −23 mV [1 mm ATP, eight cells; 1 mm glutamate, 15 cells, also tested on three cells in organotypic slices, two of which were after LPS application; 0.2 mm GABA, 15 cells, also tested on three cells in organotypic slices after LPS application; 1 mm glycine, six cells; 0.1 mm ACh, 11 cells; 0.1 mm ACPD (an mGluR agonist), eight cells; 50 μm isoproterenol (a beta adrenergic agonist), nine cells; 10 μm quinpirole (D_2_ agonist), five cells]. Of these, only ATP and isoproterenol reliably induced a current change. ATP evoked a current at −23 mV in six of the eight cells studied, which was initially outward, but became inward on repeated application of ATP. This is consistent with ATP activating both P2Y receptors (activating an outward K^+^ current) and P2X receptors (gating an inward current reversing around 0 mV) in microglia (Boucsein *et al.*, 2003), if the outward G-protein-mediated current washes out with prolonged whole-cell recording. In five out of nine cells isoproterenol induced an inward current which reversed at around 0 mV. In contrast, Färber *et al.* (2005) reported that isoproterenol and quinpirole both activated a K^+^ current in microglia in brain slices (location in the brain not specified). The lack of a response of microglia in brain slices to glutamate and GABA contradicts suggestions, based mainly on results obtained in cultured microglia, that these cells express AMPA/kainate and GABA_B_ receptors and glutamate transporters (Noda *et al.*, 1999, 2000; Kuhn *et al.*, 2004). This therefore suggests that activation of these receptors and transporters by the massive release of glutamate and GABA that occurs in ischaemia is not responsible for activating microglia in stroke.

### Kv1.3 channels are expressed in LPS-activated microglia in organotypic slices

Acute slices were incubated for up to 8 h in LPS (1 μg/mL), and no significant difference was found in the membrane properties compared with control conditions (Fig. 2C and D). The membrane resistance at negative potentials was slightly but not significantly reduced compared with control conditions (Fig. 2D inset, 0.65 ± 0.12 GΩ, *n* = 9, *P* = 0.2 compared with cells without LPS).

When organotypic slices were incubated for 24 h in LPS, the input resistance at negative potentials was also slightly but not significantly reduced (Fig. 3B inset, 0.58 ± 0.04 GΩ, *n* = 18, *P* = 0.17 compared with no LPS). However, an outward inactivating current appeared on returning to the holding potential of −23 mV after a hyperpolarizing pulse to −83 mV, because the hyperpolarization removes inactivation that is tonically present at −23 mV (Fig. 3B, arrow). This conductance was not seen in microglia in acute slices, presumably due to the shorter LPS exposure employed.

**Fig. 4:**
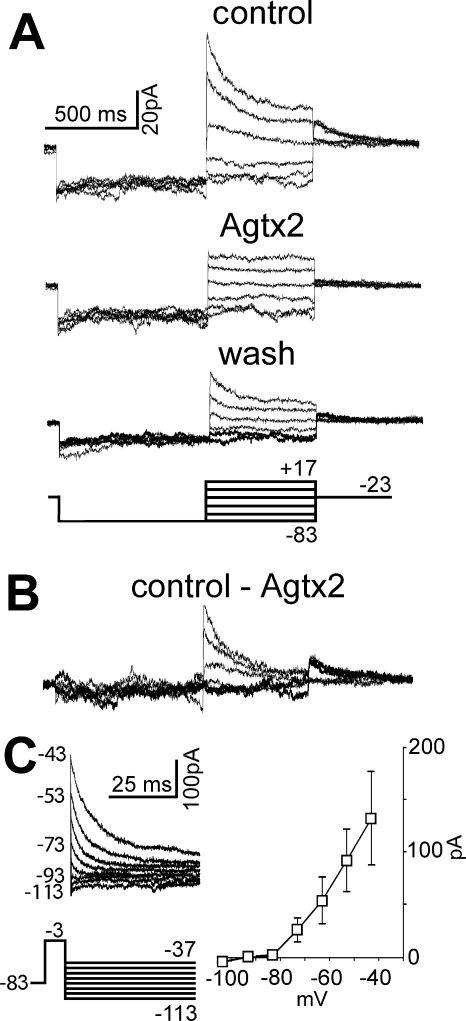
LPS-activation of microglia in organotypic slices turns on Kv1.3. (A) Microglia from organotypic slices incubated for 24 h with LPS (1 μg/mL) showed a current activated by depolarization positive to −23 mV, after removing inactivation with hyperpolarizing steps to −83 mV (inset at bottom). The current was rapidly inactivating, and was reversibly inhibited by Agitoxin-2 (2.5 nm). (B) Traces resulting from the subtraction of control and Agitoxin-2 data revealed the Agtx2-sensitive current. (C) Tail currents following a depolarizing step to -3 mV were studied to identify the reversal potential. The plot shows the voltage-dependence of the amplitude of the time-dependent tail current, which reversed around −94 mV (*n* = 3).

To characterize the outward current induced by LPS, after a hyperpolarizing pulse (to −83 mV) to remove inactivation, depolarizing voltage steps in 20-mV increments were applied. The transient outward current was observed at potentials positive to −40 mV (Fig. 4A). Agitoxin-2 (5 nm) blocked the time-dependent conductance (Fig. 4A and B, *n* = 4), suggesting that the channels responsible are Kv1.3 (Grissmer *et al.*, 1994; Newell & Schlichter, 2005). To test whether the current was carried by K^+^ ions, a depolarizing pulse to −3 mV was applied from −83 mV to activate the current, and the amplitude of the time-dependent tail current seen on repolarizing to different negative voltages was plotted against the voltage. The tail currents reversed at −94 ± 6 mV (*n* = 3), near to the K^+^ equilibrium potential (*E*_K_ = −104 mV), suggesting that this is a K^+^ conductance (Fig. 4C). The appearance of Kv1.3 current in organotypic slices after LPS incubation is in agreement with previous reports from microglial cells in culture (Norenberg *et al.*, 1992; Fordyce *et al.*, 2005; Newell & Schlichter, 2005). In microglia from acute slices a similar current was described by Boucsein *et al.* (2000) in response to facial nerve axotomy.

### Microglia in acute and organotypic slices express a current activated by depolarization that is not carried by protons or K^ +^

**Fig. 5:**
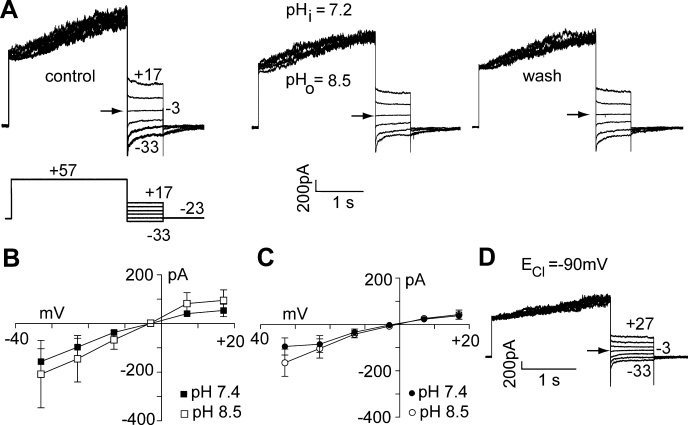
The time-dependent outward current is not carried by H^+^ or Cl^−^. (A) The reversal potential of the current activated at +57 mV (in a cell in an organotypic slice, pH_i_ = 7.2, holding potential −23 mV) is identified by the tail currents produced on repolarizing (inset at bottom) to voltages between −33 and +17 mV (left panel). The reversal potential did not change when the external solution pH was switched to 8.5 (middle panel; right panel shows responses after washing to pH_o_=7.4 solution). (B) Mean *I–V* relationship of tail currents measured at pH_o_ 7.4 (filled squares, *n* = 11) and 8.5 (open squares, *n* = 6) in acute slices. The reversal potential was near 0 mV in both solutions. (C) Similarly, in organotypic slices the reversal potential was near 0 mV at pH_o_ 7.4 (filled circles, *n* = 9) and pH 8.5 (open circles, *n* = 7). (D) Using a low [Cl^−^] internal did not affect the reversal potential of the current (either with pH_o_ = 7.4 as here, or 8.5: data not shown), ruling out Cl^−^ as a permeant ion (data from a cell in an organotypic slice). Arrows indicate the reversal potential of the current.

The large outward current activated by depolarization from −23 mV was observed in acute and cultured slices in both resting and LPS-activated microglia (Figs 2 and 3). Its properties (activation, amplitude and kinetics) were reminiscent of the proton current described in rat cultured microglia (Eder *et al.*, 1995). To study the ion movements underlying the conductance, a voltage step to +57 mV was applied to activate the current, followed by steps back to between −33 and +17 mV. The reversal potential of the time-dependent current decay seen on repolarization as the current deactivates was not significantly different (*P* = 0.94) in acute and organotypic slices, being −2.4 ± 1.5 mV (*n* = 11) and −2.5 ± 0.9 mV (*n* = 9) respectively (Fig. 5A–C). From the Nernst equation, the equilibrium potential for H^+^ ions with an internal pH (pH_i_) of 7.2 and an external pH (pH_o_) of 7.4 is −12 mV, not far from the measured reversal potential, suggesting that the time-dependent current could be carried by protons. However, when pH_o_ was increased to 8.5, bringing the equilibrium potential for H^+^ to −79 mV, the reversal potential of the current did not change significantly, being −3.1 ± 1.0 mV (*n* = 6, *P* = 0.73, compared with pH_o_=7.4) in acute, and −2.6 ± 1.1 mV (*n* = 7, *P* = 0.95 compared with pH_o_=7.4) in organotypic slices (Fig. 5A–C), suggesting that H^+^ is not the main ion permeating these channels. The same experiment was also performed in organotypic slices using TAPS instead of HEPES to buffer the external solution at pH 8.5. Similarly, no significant shift in the reversal potential was observed, as the current reversed at −0.95 ± 0.80 mV (*n* = 4, *P* = 0.35 compared with the value of −2.5 ± 0.9 mV at pH_o_=7.4), confirming the previous result (data not shown). To rule out further the possibility that H^+^ ions mediate this conductance, pH_i_ was decreased to 6.45, a manoeuvre that is reported to increase activation of the proton current (Schilling *et al.*, 2002), and which will shift the equilibrium potential for H^+^ to −58 mV at pH_o_=7.4 and to −125 mV at pH_o_=8.5 (this external solution was buffered by TAPS instead of HEPES). With these solutions, the size of the time-dependent current was not increased [on depolarizing to +57 mV the time-dependent increase in outward current was 327 ± 85 pA in six cells, not significantly different (*P* = 0.43) from the 484 ± 162 pA measured in seven cells with pH_i_=7.2], and the reversal potential of the current activated by the step to +77 mV did not shift significantly (*P* = 0.42), being −1.1 ± 2.3 mV (*n* = 6) at pH_o_ 7.4 and −3.2 ± 0.9 mV (*n* = 6) at pH_o_ 8.5. These data rule out the possibility that H^+^ ions play a major part in mediating this conductance.

The *I–V* relationship of the tails produced on repolarization after activating the time-dependent outward current (Fig. 5A–C) has a reversal potential close to the predicted value of *E*_Cl_ for the solutions used (−5.2 mV). To test whether Cl^−^ is a permeant ion mediating this conductance, a low Cl^−^ intracellular solution was used (see Materials and methods), which brought the equilibrium potential for Cl^−^ to −90 mV. However, the reversal potential of the time-dependent outward current did not change significantly compared with control (*P* = 0.2), being 1.7 ± 4.0 mV (*n* = 5), ruling out the possibility that the current is carried by Cl^−^ (Fig. 5D).

We conclude that this depolarization-activated conductance in microglia from acute and organotypic slices is distinct from the proton current described in cultured microglia, is not carried solely by K^+^ or Cl^−^ and has not been previously described in the literature, so from now on we refer to it as a ‘novel’ non-specific cation conductance (reflecting the fact that it is not mediated by the main anion present, and reverses around 0 mV and so cannot be carried solely by Na^+^, K^+^ or Ca^2+^).

The amplitude of this current was similar in organotypic and acute slices. The time-dependent current increase (measured from 0 to 2 s of a depolarizing step to +57 mV) was 484 ± 162 pA (*n* = 7) in acute slices and 604 ± 244 pA (*n* = 5) in organotypic slices (not significantly different, *P* = 0.68). The current magnitude was not affected by activation by LPS in organotypic slices: after LPS treatment it was 406 ± 77 pA (*n* = 8; not significantly different from with no LPS treatment, *P* = 0.37). As the shape and size of microglia also differ somewhat between acute and organotypic slices, and between organotypic slices without and with LPS exposure (see above) we also quantified the size of the current normalized by cell capacitance, but this also showed no significant difference between any of these three conditions [33.9 ± 8.9 pA/pF in acute slices, 30.2 ± 8.6 pA/pF in organotypic slices (*P* = 0.78 compared with acute slices) and 31.2 ± 9.5 pA/pF in organotypic slices with LPS (*P* = 0.94 compared with no LPS)]. Similarly, the time course of the current tail decay seen on repolarizing to −23 mV after activating the outward current was not significantly different between acute and cultured slices and was not affected by LPS. Fitting a single exponential to the decay in cells in acute slices gave a time constant of 293 ± 57 and 320 ± 70 ms in 20 control cells and 10 LPS-treated cells (not significantly different, *P* = 0.78), respectively, and in organotypic slices gave a time constant of 246 ± 69 ms (*P* = 0.61 compared with acute slices) and 271 ± 52 ms in 23 control cells and 12 LPS-treated cells (not significantly different, *P* = 0.81), respectively.

### Comparison of the novel conductance with ADP ribose-gated TRPM2 channels

A candidate for generating the depolarization-activated non-specific conductance is the TRPM2 subfamily of TRP channels (for a review see Fleig & Penner, 2004), which have a reversal potential near 0 mV and a linear *I–V* relationship like the novel conductance described here (Fig. 5B and C). In macrophages TRPM2 channels generate a non-specific cation current in response to depolarization or intracellular dialysis of ADP ribose (Campo *et al.*, 2003), and internal ADP ribose also generates a cation current in cultured microglia (Kraft *et al.*, 2004).

**Fig. 6:**
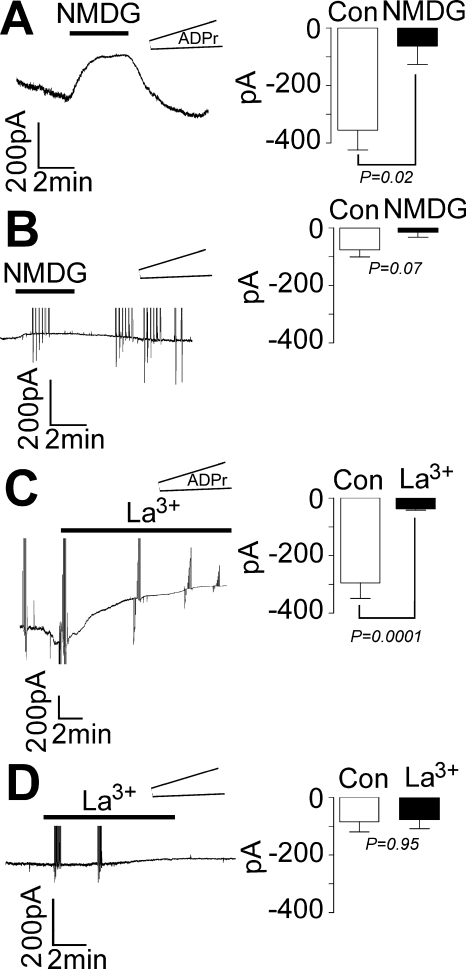
Internal ADP ribose induces a La^3+^-sensitive cation current. (A) Top trace: at −23mV, ADP ribose (100 μm) introduced from the pipette induced an inward current that was almost abolished when NMDG replaced Na^+^ in the external solution. Mean data on the right show the membrane current 3–4 min after entering whole-cell mode (Con) and the membrane current after replacing external Na^+^ with NMDG (*n* = 4). (B) No such current developed when there was no ADP ribose in the pipette. Right bars are membrane current 4 min after entering whole-cell mode (Con) and the membrane current after replacing external Na^+^ with NMDG (*n* = 7). (C) The ADP ribose current was blocked by 100 μm La^3+^ [mean data on the right show membrane current 4 min after entering whole-cell mode (Con) and the membrane current after adding external La^3+^ (*n* = 4)]. (D) La^3+^ does not suppress such a large current in the absence of ADP ribose. Mean data on the right are as in C. Data in A and B are from organotypic slices, data in C and D are from acute slices. Data traces shown are after some minutes in whole-cell mode. Vertical lines on some traces are voltage steps used to study voltage-activated currents or to check pipette series resistance.

To test the possibility that the novel current is due to TRP channels, we first demonstrated the presence of an ADP ribose-evoked current in microglia. When ADP ribose (100 μm) was present in the pipette solution (with ATP omitted from the solution as it has been reported to inhibit ADP ribose-evoked currents: Campo *et al.*, 2003) an inward current developed at the resting potential, which was inhibited when the external Na^+^ was replaced by the large cation *N*-methyl-d-glucamine (NMDG: Fig. 6A, *n* = 4, *P* = 0.02, cells in organotypic slices). When ADP ribose was omitted from the pipette solution, the inward current reached 4 min after going to whole-cell mode was five-fold smaller (*P* = 7 × 10^−5^, Fig 6B), and replacing Na^+^ with NMDG no longer significantly reduced the current (*P* = 0.07, *n* = 7, Fig. 6B). Similarly, in microglia in acute slices, the inward current reached after 4 min in whole-cell mode was much larger with ADP ribose in the pipette (275 ± 35 pA, *n* = 8) than without ADP ribose (66 ± 13 pA, *n* = 16; significantly different, *P* = 7 × 10^−7^; data not shown).

**Fig. 7:**
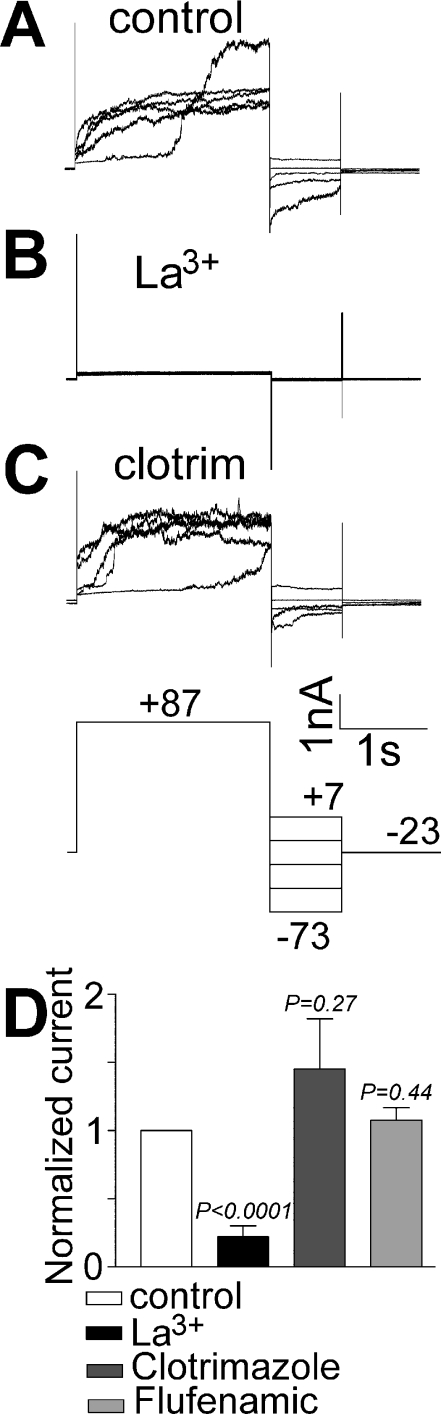
Effect of La^3+^, clotrimazole and flufenamic acid on the outward current. (A–C) The current evoked by a positive step from −23 to +87 or +77 mV in normal solution (A) was inhibited by 100 μm La^3+^ (B), but was not affected by 30 μm clotrimazole (C) or 200 μm flufenamic acid (data not shown). Data in A–C are from the same cell. (D) Mean effect of La^3+^ (*n* = 7), clotrimazole (*n* = 5) and flufenamic acid (*n* = 6) on the time-dependent current evoked by the depolarizing step (the amplitude was measured by averaging the five traces of each trial, and taking the difference in current between the beginning and the end of the voltage steps to +87 mV for La^3+^ and clotrimazole, and to +77 mV for flufenamic acid); data are normalized to the size of the current in control solution in the same cell. *P* values compared with control are < 0.0001, 0.13 and 0.22 for La^3+^, clotrimazole and flufenamic acid, respectively.

Lanthanum (La^3+^, 100 μm) was found to inhibit the inward current recorded with ADP ribose in the pipette (Fig. 6C, *n* = 4, *P* = 0.0001), but did not significantly inhibit the membrane current in the absence of internal ADP ribose (Fig. 6D, *P* = 0.95, *n* = 3). La^3+^ also inhibited the outward current activated by depolarization to +87 mV (Fig. 7A, B and D: *n* = 7, *P* = 0.0001). This is similar to what was found by Campo *et al.* (2003) for the ADP ribose- and depolarization-activated conductances in macrophages (although in our experiments a lower lanthanum concentration was more effective), but disagrees with the finding of Kraft *et al.* (2004) that La^3+^ did not block the ADP ribose-activated current in cultured rat microglia.

Neither clotrimazole (30 μm), an antifungal agent known to block TRPM2 channels (Hill *et al.*, 2006), nor flufenamic acid (200 μm), which also blocks these channels (Hill *et al.*, 2004), blocked the outward current activated by depolarization (Fig. 7C and D).

If ADP ribose dialysis and depolarization are alternative ways to activate the same conductance, as suggested by Campo *et al.* (2003), then having ADP ribose in the pipette ought to occlude the generation of the depolarization-evoked conductance, as found by Campo *et al.* (2003) in macrophages. We found, however, that having ADP ribose in the pipette did not reduce the outward current activated when microglial cells in acute slices were depolarized to +67 mV, and in fact the mean current showed an increase (non-significant, *P* = 0.11): the average current at +67 mV in control solution was 484 ± 162 pA (*n* = 7), and with intracellular ADP ribose was 895 ± 170 pA (*n* = 6). This suggests that the two currents are mediated by different channels.

In summary, these data are not consistent with the outward current being generated by TRPM2 channels.

### Imaging confirmation of NADPH oxidase activity

To check if the NADPH oxidase is active in microglia in slices, ROS production was measured with confocal microscopy using dihydroethidium. Organotypic slices 10 days old were chosen for this experiment as microglial cells are in the resting state by this stage (Duport & Garthwaite, 2005), allowing us to detect an increase in ROS production when phorbol myristate acetate (PMA, 100 nm) was added (Dana *et al.*, 1994; in acute slices microglial activation by the slicing procedure might result in the NADPH oxidase already being turned on). No preincubation with LPS was used. Fluorescence was measured at intervals of 5 s in control solution to obtain a baseline measurement, and subsequently in PMA to test whether there was an increase in fluorescence intensity due to ROS production when the NADPH oxidase is activated by the PMA (Dana *et al.*, 1994). Finally, in the presence of PMA the NADPH oxidase blocker diphenyliodonium (DPI, 30 μm) was added to check that ROS production decreased.

**Fig. 8:**
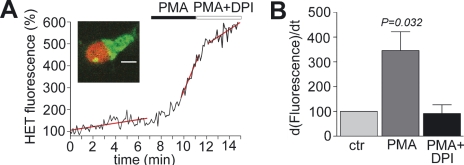
Measurement of microglial ROS production with dihydroethidium fluorescence. (A) Fluorescence intensity measured from a microglial cell (inset, scale bar 10 μm) in an organotypic slice, plotted normalized to 100% as the initial value. Dihydroethidium showed an increased rate of oxidation (red line) after exposure to PMA (0.1 μm). DPI (30 μm) decreased the oxidation rate to baseline level. (B) The rate of increase of fluorescence in the different solutions (change of fluorescence over the last 200 s in each solution) in five cells, normalized so that the rate of increase in normal solution (ctr) is defined as 100%.

The plot in Fig. 8A shows that PMA induced an increase in ROS production, seen as an increase in the slope of the dihydroethidium fluorescence curve compared with control (the increase in fluorescence over the last 200 s of application of each solution was measured, and normalized to the value obtained in control solution). On application of DPI, the slope of the curve decreased, consistent with the NADPH oxidase being inhibited. These data are quantified for five cells in three slices in Fig. 8B.

### The relative importance of the novel outward current and Kv1.3 for charge compensation

**Fig. 9:**
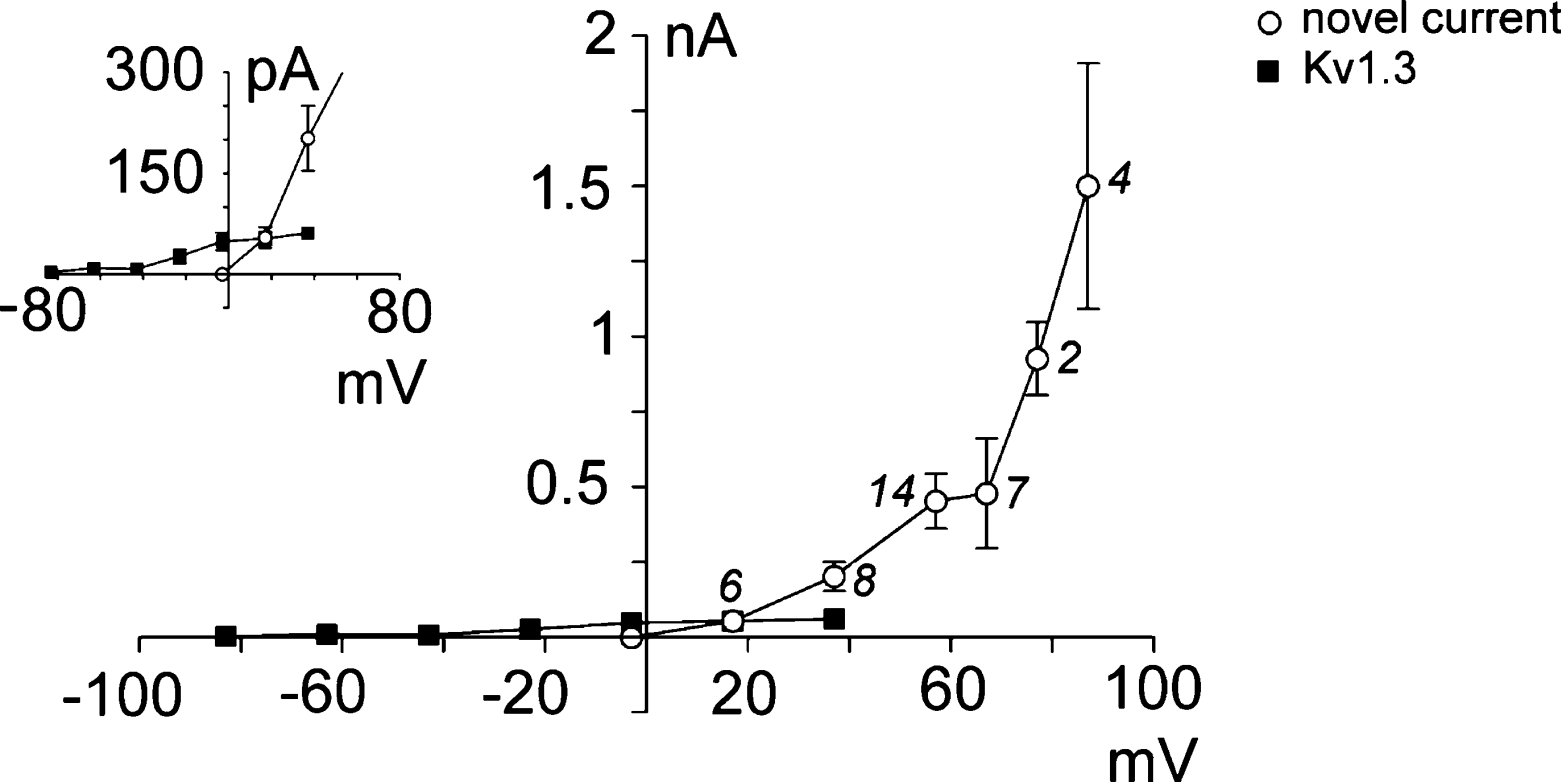
Relative contribution of Kv1.3 and the novel current to charge compensation. The graph plots the steady-state outward current carried by Kv1.3 (defined by its Agitoxin-2 sensitivity) and the amplitude of the time-dependent outward current generated by the novel conductance, as a function of voltage. Up to +20 mV the capacity to generate an outward current to compensate for NADPH oxidase charge movement is larger for Kv1.3 (filled squares, *n* = 4), while at more positive potentials the novel conductance plays the major role (open circles, the numbers next to the symbols indicate the number of cells studied for each potential). Inset is an expanded version of the region near 0 mV.

In LPS-activated cells, both Kv1.3 and the novel conductance generating the time-dependent non-specific cation current could act as a charge-compensating mechanism for NADPH oxidase activity. To measure the relative contribution of Kv1.3 and the novel conductance, the amplitudes of the currents were measured as a function of voltage (Fig. 9, *n* = 4 for Kv1.3; *n* = 2–14 at different potentials for the novel conductance, pooling data from acute and organotypic slices as they did not differ significantly). The steady-state amplitude of the current generated by Kv1.3 was defined as the current blocked by agitoxin at each voltage. For the novel conductance, we measured the time-dependent outward current 2 s after stepping to each voltage – as the current was still increasing at this time the true steady-state current would be larger.

At potentials more negative than +20 mV, Kv1.3 acted as the main charge-compensating mechanism, while at more positive potentials the novel conductance was by far the most effective means of extruding the net positive charge accumulated inside the cell by NADPH oxidase activity. Analogously, in neutrophils Rada *et al.* (2005) concluded that although at negative potentials K^+^ currents would be the main charge-compensating mechanism, at positive potentials H^+^ currents dominate.

## Discussion

The importance of a mechanism in microglia to compensate for the charge moved by the NADPH oxidase can be seen as follows. Morgan *et al.* (2003) measured an NADPH oxidase current of ∼30 pA in human eosinophils. Assuming the same value for rat microglia, and an estimated membrane resistance of ∼2.1 GΩ (see Results), and ignoring the decrease in resistance produced at depolarized potentials by the presence of the Kv1.3 conductance or the novel conductance, this would depolarize the cell to about +21 mV from an estimated resting potential of −42 mV. The true depolarization might be much larger because our correction of the input resistance for the presence of the seal between the cell and the electrode may not be accurate if the seal resistance alters during the transition to whole-cell mode. Indeed, non-invasive measurements in cultured microglia have suggested a true input resistance of ∼8 GΩ (Newell & Schlichter, 2005), which, if appropriate for microglia in slices, would imply a substantially more negative resting potential than we measure and, in the absence of voltage-gated channel activation, an oxidase-induced depolarization of about +240 mV from that resting potential. Depolarization beyond 0 mV inhibits the NADPH oxidase, with 50% inhibition occurring at about +100 mV (DeCoursey *et al.*, 2003), making it essential for a charge compensation mechanism to exist if killing of cells by microglial release of reactive oxidative species is to occur.

It is generally assumed that either voltage-gated H^+^ channels (DeCoursey *et al.*, 2003), voltage- or calcium-activated K^+^ channels (Khanna *et al.*, 2001; Reeves *et al.*, 2002; Ahluwalia *et al.*, 2004; Fordyce *et al.*, 2005; Segal, 2005), or Cl^−^ channels (Thomas *et al.*, 2007) generate outward current to compensate for the charge movement produced by the NADPH oxidase. We were surprised to find therefore that microglia *in situ* in slices (both acute and cultured, and whether activated by LPS or not) do not show a significant H^+^ current (Fig. 5). This is not due to our choice of pH buffer concentration in the pipette, because others (DeCoursey, 1991;Eder *et al.*, 1995; McLarnon *et al.*, 1997) have reported H^+^ currents in cultured epithelial cells and microglia with pH buffer concentration similar to that which we employ (they saw > 100 pA H^+^ currents with 5–20 mm MES, 17 mm PIPES or 10 mm HEPES in the pipette). Furthermore, although microglia in slices do show a Kv1.3 channel that is turned on when the cells become activated (Fig. 4), this generates only a small part of the possible charge compensation current at potentials positive to +20 mV (Fig. 9). Instead, the majority of the charge compensation at potentials positive to +20 mV is probably performed by a novel depolarization-activated conductance, with a reversal potential of ∼0 mV (Fig. 5), that is not carried by H^+^, nor by Cl^−^, nor exclusively by K^+^ (because of the reversal potential) and is presumably a non-selective cation current. This current is blocked by La^3+^, as are depolarization- and ADP ribose-activated TRP channels reported previously in macrophages (Campo *et al.*, 2003), but this current in microglia was not blocked by clotrimazole or flufenamic acid, which block TRPM2 channels.

There is a striking contrast between our recordings, which show no depolarization-activated proton current, and previous work on cultured microglia, which routinely reports such currents (McLarnon *et al.*, 1997; Eder & DeCoursey, 2001). We attribute this difference, and the relative unimportance of K^+^ and Cl^−^ channels in generating an outward current at positive potentials in our cells, to the culture conditions used for pure microglial cultures altering the ion channels which are expressed. We cannot rule out the possibility, however, that (as suggested by Thomas *et al.*, 2007) the particular stimulus used to activate microglia may determine which ion channel types are upregulated to mediate charge compensation. Nevertheless, our results demonstrate that microglia in cultured slices (in which the microglia are surrounded by astrocytes and neurons) are apparently identical to those in acutely cut slices, suggesting that organotypic slices may offer a useful method to study microglia which retain a phenotype similar to microglia *in situ*, yet can be studied in longer term experiments than is possible in acute slices.

The absence of proton channels in microglia *in situ* raises the question of how the H^+^ liberated intracellularly by the NADPH oxidase is removed from the cell. For an electron current of ∼30 pA, as in eosinophils (Morgan *et al.*, 2003), and considering the volume of the microglia to be equivalent to that of a sphere with a radius of 7.5 μm, the oxidase would produce 170 μmol/L of H^+^ per second, which (for a buffering power of 20 mm/pH unit) would decrease the pH_i_ by ∼0.5 U/min. This potential pH change would need to be accommodated by the Na^+^/HCO_3_^−^ cotransporters and Na^+^/H^+^ exchangers which regulate pH_i_ in microglia (Faff *et al.*, 1996; Ohlemeyer & Kettenmann, 2001). Maximal Na^+^-dependent H^+^ fluxes have been demonstrated to be equivalent to a current of > 20 pA in (slightly larger) Chinese hamster ovary fibroblasts (Fuster *et al.*, 2004), so Na^+^/H^+^ exchangers alone could suffice to minimize the cytoplasmic pH change produced by NADPH oxidase activity.

In summary, our data suggest that for microglia *in situ*, as opposed to in culture, a previously undescribed non-specific cation current may mediate charge compensation for the electron-extruding activity of NADPH oxidase.

## Abbreviations

DIV: days *in vitro*
DPI: diphenyliodonium
LPS: lipopolysaccharide
NMDG: *N*-methyl-d-glucamine
P: postnatal day
PMA: phorbol myristate acetate
ROS: reactive oxygen species
TRPM2: transient receptor potential melastatin2
